# Impact of emphysema on biomarkers in hospitalized COPD patients with community-acquired pneumonia: a retrospective study

**DOI:** 10.1186/s12890-026-04364-2

**Published:** 2026-05-23

**Authors:** Rabia Yurt, Meltem Agca, Ayla Turkar, Eylem Tuncay, Sinem Gungor, Nazli Huma Teke, Baran Gundogus, Sibel Arinc, Ipek Ozmen

**Affiliations:** 1 Department of Pulmonology, University of Health Sciences, Sureyyapasa Chest Diseases and Thoracic Surgery Training and Research Hospital, Istanbul, Turkey; 2 Department of Radiology, Umraniye Training and Research Hospital, Istanbul, Turkey; 3 Department of Pulmonology, University of Health Sciences, Sancaktepe Prof. Dr. Ilhan Varank Training and Research Hospital, Istanbul, Turkey; 4 Department of Pulmonology, University of Health Sciences, Basaksehir Cam and Sakura City Hospital, Istanbul, Turkey

**Keywords:** COPD, Emphysema, Biomarkers, Pneumonia, C-Reactive Protein, Procalcitonin

## Abstract

**Background:**

Chronic obstructive pulmonary disease (COPD) is a heterogeneous disorder in which chronic bronchitis and emphysema coexist to varying degrees. Emphysema has distinct clinical and prognostic features. C-reactive protein (CRP), procalcitonin (PCT), white blood cell count (WBC), and neutrophil values are used for diagnosing community-acquired pneumonia (CAP). This study investigated whether these biomarkers differ in emphysematous patients with CAP.

**Methods:**

Patients with COPD hospitalized with CAP between January 2016 and February 2023 were divided into emphysema and non-emphysema groups based on radiologic findings. Emphysema subtypes were classified, and quantitative Goddard scoring was performed. Admission demographic, clinical and laboratory data, hospital stay length and clinical outcomes (discharge, intensive care unit [ICU] transfer, in-hospital mortality) were compared between groups. CRP and PCT levels were dichotomized as low and high using predefined thresholds (CRP ≤ 200 vs. >200 mg/L; PCT < 0.10 vs. ≥0.10 µg/L). The independent effect of emphysema on low CRP and PCT levels was assessed using multivariable binary logistic regression analysis.

**Results:**

Of the 135 patients included, 71 (53%) had emphysema. Median CRP and PCT levels were significantly lower in the emphysema group than in the non-emphysema group (CRP: 116, interquartile range [IQR] 56–187 vs. 254 (IQR 213-310 mg/L); *p* < 0.001; PCT: 0.132 [ IQR 0.079–0.536] vs. 0.356 [IQR 0.171–1.660] µg/L; *p* = 0.007, respectively). Median WBC and neutrophil counts were also lower (*p* = 0.036; *p* = 0.035, respectively). In multivariable analysis, emphysema (odds ratio [OR] 54.274; 95% Confidence Interval [CI] 14.545–202.526; *p* < 0.001), higher glomerular filtration rate (OR 1.021; 95% CI 1.002–1.040; *p* = 0.026), and prior antibiotic use (OR 4.278; 95% CI 1.199–15.264; *p* = 0.025) were independently associated with low CRP. For low PCT, emphysema remained the only significant predictor (OR 3.694; 95% CI 1.145–11.921; *p* = 0.029). Length of stay was longer in emphysema (9 vs.7 days; *p* = 0.010), with no differences in ICU transfer or mortality.

**Conclusion:**

In patients with emphysema, inflammatory biomarkers at CAP diagnosis were lower compared with those without emphysema. These findings suggest a potential difference in biomarker response; however, the retrospective design limits causality. Further prospective studies are needed to confirm these results and clarify their clinical relevance.

**Trial registration:**

Not applicable. This study is a retrospective observational analysis and was not registered as a clinical trial.

## Background

Chronic obstructive pulmonary disease (COPD) is a major public health problem associated with increasing morbidity and mortality worldwide and is projected to become the third leading cause of death by 2030 [1]. Within its heterogeneous structure, in which chronic bronchitis and emphysema coexist to varying degrees, emphysema is recognized as a distinct phenotype with specific clinical and prognostic characteristics [2, 3].

Pneumonia is one of the most lethal infectious diseases globally [4, 5], and COPD is among its most common comorbidities [6]. Structural airway deformation, impaired immune responses, mucus retention, and the use of inhaled corticosteroids in COPD may increase the risk of infection and predispose patients to pneumonia [7, 8]. In addition, the loss of lymphoid follicles has been associated with increased susceptibility to respiratory infections [9]. Previous studies have demonstrated that the clinical course and inflammatory response of pneumonia in patients with COPD differ from those in patients without COPD [10, 11], and the presence of COPD has been reported to increase pneumonia-related morbidity and mortality [12].

The most commonly used biomarkers in the diagnosis of pneumonia include C-reactive protein (CRP), procalcitonin (PCT), white blood cell (WBC) count, and neutrophil count [13], and studies have shown that these markers may help distinguish COPD exacerbations from pneumonia [14, 15]. However, studies directly comparing biomarker profiles between emphysema and non-emphysema phenotypes among COPD patients with pneumonia are limited, and the effect of emphysema on the inflammatory response remains insufficiently understood.

Therefore, the aim of this study was to compare CRP, PCT, WBC, and neutrophil levels between computed tomography (CT)–confirmed emphysema and non-emphysema phenotypes in COPD patients diagnosed with community-acquired pneumonia (CAP) and to evaluate the impact of emphysema on these biomarkers.

## Materials and methods

### Study design and ethics

This study was designed as a single-center, retrospective, observational, cross-sectional cohort study and was conducted at a tertiary referral chest diseases hospital. All data were obtained from the hospital’s electronic medical records. Ethical approval was granted by the Ethics Committee for Non-Interventional Clinical Research (protocol code: 116.2017.R-286).

### Patient selection

Medical records of 135 patients with COPD who were admitted to the chest diseases clinic with a diagnosis of pneumonia between January 2016 and February 2023 were retrospectively reviewed. Thoracic CT and/or high-resolution computed tomography (HRCT) scans were used to classify patients into two groups: those with emphysema and those without emphysema. CT images used to establish the diagnosis of emphysema were obtained within 1 year before or after the diagnosis of pneumonia. The CT scans obtained during periods with active pneumonia findings (such as consolidation, atelectasis, or pleural effusion) were not used for emphysema scoring in any patient. Emphysema assessment was performed using thoracic CT scans acquired within up to 1 year prior to hospital admission in most patients. In a lower proportion of patients, follow-up CT scans obtained after completion of pneumonia treatment were used for emphysema scoring. In this way, the potential impact of pneumonia-related parenchymal changes on the evaluation of underlying emphysema was minimized.

Demographic characteristics (age, sex, smoking history, and comorbidities), clinical symptoms (fever, dyspnea, cough, sputum production, and pleuritic chest pain), radiological findings of pneumonia (localization and presence of pleural effusion), medical treatments administered during hospitalization, oxygen support modalities (room air, nasal oxygen, and non-invasive mechanical ventilation [NIMV]), length of hospital stay, clinical outcomes (discharge and transfer to the intensive care unit [ICU]), and in-hospital mortality were compared between the two groups. Laboratory parameters, including biomarkers (CRP and procalcitonin), complete blood count and biochemical analyses, were also compared between the groups. For patients with recurrent hospital admissions, only one medical record that met the study inclusion criteria was selected.

In our study, all biomarkers were measured at the time of hospital admission as part of the routine initial clinical and laboratory evaluation. Blood samples were obtained upon admission, before treatment initiation, ensuring a standardized time point across all patients.

### COPD diagnosis

The institution where the study was conducted is a tertiary referral specialty hospital receiving patients from surrounding provinces. For the diagnosis of COPD, the following criteria were considered: clinically compatible COPD symptoms, CT findings consistent with COPD, documented COPD medication reports issued by a chest diseases specialist in the hospital’s electronic medical records, and regular use of bronchodilators.

In our institution, spirometry is routinely performed for all patients with suspected COPD, and the diagnosis is typically based on a post-bronchodilator FEV₁/FVC ratio < 70% [2].

### Emphysema diagnosis

Thoracic CT or HRCT images archived in the hospital’s picture archiving and communication system (PACS) were used for emphysema diagnosis and severity scoring. All scans were performed in the supine position using a 16-slice multislice CT scanner (Toshiba Alexion 16; Toshiba Medical Corporation, Tokyo, Japan) and were obtained during full inspiration. Slice thickness was 4 mm for standard thoracic CT and 1 mm for HRCT. Images were reviewed in lung window settings and in random order during a dedicated reading session. On CT imaging, areas of low attenuation with well-defined borders compared with adjacent normal lung parenchyma, with or without wall thickness < 1 mm, as well as multiple blebs larger than 1 cm, were considered radiological features of emphysema [16].

Emphysema subtypes were classified into four categories: centriacinar, mixed (centriacinar plus paraseptal), panacinar, and paraseptal.

**Fig. 1:**
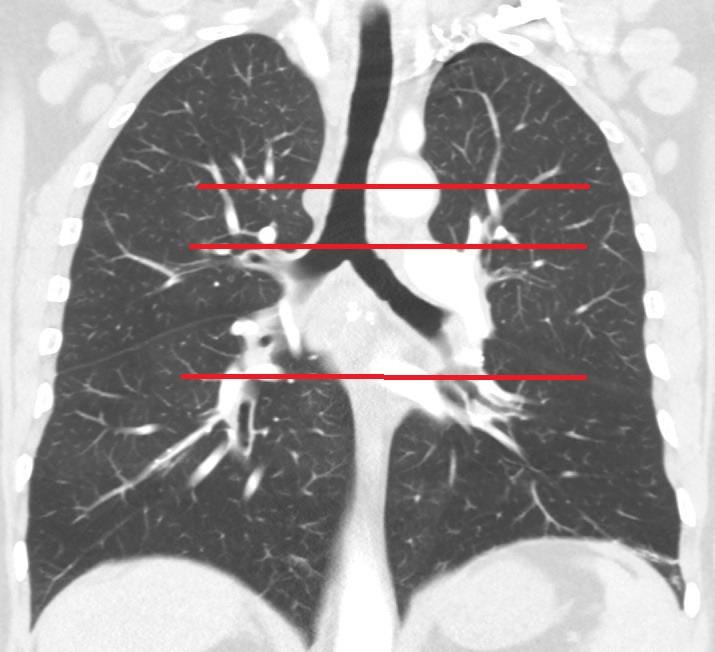
Goddard emphysema scoring uses aortic arch, carina, inferior pulmonary veins, plus both lungs: six regions

The Goddard scoring system was used to assess emphysema severity [17]. Axial images at three anatomical levels—the aortic arch, the carina, and the level of the inferior pulmonary veins—were selected to divide the lungs into six regions (right and left lungs at each level) (Fig. 1).

Each region was scored based on the percentage of emphysema involvement as follows: 1%–25% = 1 point; 26%–50% = 2 points; 51%–75% = 3 points; and 76%–100% = 4 points. The total score, obtained by summing the six regional scores, ranged from a minimum of 1 to a maximum of 24. Emphysema severity was categorized as mild (1–6), moderate (7–12), moderate-to-severe (13–18), and severe (19–24).

Emphysema presence and severity were assessed visually. The presence of emphysema was evaluated by a thoracic radiologist and a pulmonologist. Emphysema severity was assessed using a structured semi-quantitative visual scoring system based on predefined anatomical levels and percentage involvement. Quantitative CT software metrics were not used.

The radiologist was blinded to all clinical and laboratory data during CT interpretation.

The presence of emphysema (yes/no) was independently evaluated on thoracic CT scans by one experienced thoracic radiologist and one pulmonologist. No discrepancies were observed between the two reviewers regarding the presence of emphysema. Interobserver agreement for the presence of emphysema between the thoracic radiologist and the pulmonologist was excellent (Cohen’s κ = 0.87, 95% CI 0.80–0.93, *p* < 0.001).

### Pneumonia diagnosis

Pneumonia was diagnosed based on the presence of: (a) a new infiltrate on chest radiography or CT; (b) one or more clinical signs or symptoms suggestive of pneumonia, including cough, sputum production, dyspnea, fever, or chest pain; and (c) exclusion of alternative diagnoses [4].

The Pneumonia Severity Index (PSI) and CURB-65 scores were used to assess pneumonia severity and prognosis. The PSI score incorporates age, sex, nursing home residence, comorbid conditions, vital signs, laboratory findings, chest radiograph results, and oxygenation status [18]. The CURB-65 score includes confusion, blood urea nitrogen > 7 mmol/L, respiratory rate ≥ 30 breaths/min, systolic blood pressure < 90 mmHg or diastolic blood pressure ≤ 60 mmHg, and age ≥ 65 years; each criterion is assigned one point [19].

Pneumonia severity was assessed at hospital admission using the CURB-65 and PSI scores for all patients.

### Biomarkers and laboratory findings

#### CRP and PCT

CRP was dichotomized using a cut-off value of 200 mg/L; values ≤ 200 mg/L were classified as low CRP, and values > 200 mg/L as high CRP [20].

For PCT, the cut-off value was determined as 0.10 µg/L in accordance with the manufacturer’s reference range; values < 0.10 µg/L were classified as low PCT, and values ≥ 0.10 µg/L as high PCT [21].

Procalcitonin (PCT) levels were measured using a highly sensitive, time-resolved amplified cryptate emission (TRACE) technology assay (PCT Kryptor^®^; BRAHMS AG, Hennigsdorf, Germany), with a lower detection limit of 0.02 ng/mL (values below this limit were assigned a value of zero) and a functional sensitivity of 0.06 ng/mL.

For interpretation, PCT values < 0.1 µg/L are not indicative of any infection; 0.1–0.5 µg/L indicate localized bacterial infection risk; 0.5–2 µg/L indicate systemic bacterial infection risk; 2–10 µg/L indicate severe systemic bacterial infection; and values > 10 µg/L indicate severe systemic bacterial infection.

GFR was calculated for all patients. Renal function was classified according to the National Kidney Foundation (NKF) guidelines into five stages: Stage 1, GFR ≥ 90 mL/min/1.73 m² (normal kidney function); Stage 2, GFR 60–89 mL/min/1.73 m² (mildly decreased); Stage 3, GFR 30–59 mL/min/1.73 m² (mild to moderately decreased); Stage 4, GFR 15–29 mL/min/1.73 m² (severely decreased); and Stage 5, GFR < 15 mL/min/1.73 m² (kidney failure) [22].

### Inclusion criteria

- Patients with a diagnosis of COPD and CAP.

### Exclusion criteria

**Fig. 2:**
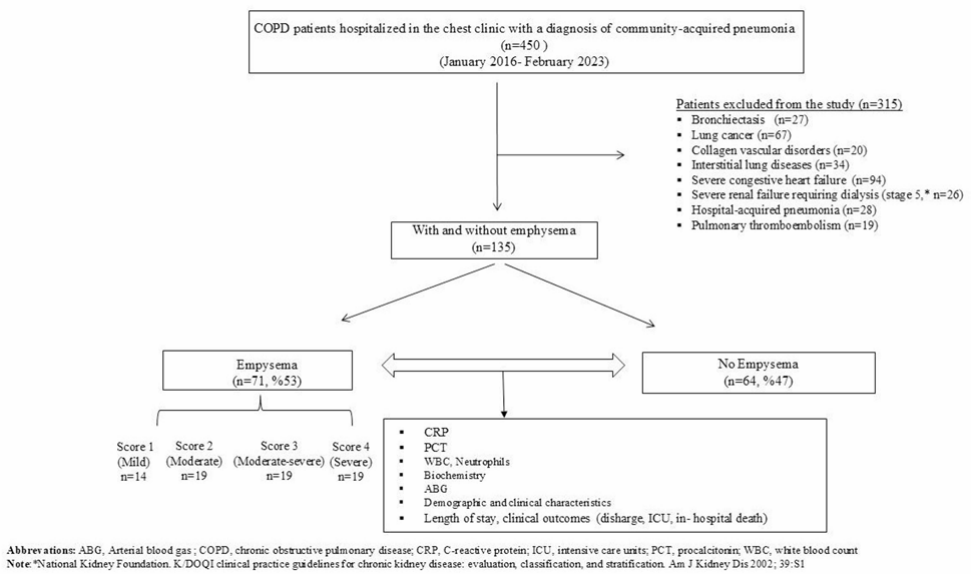
Flowchart

- Absence of thoracic CT or HRCT imaging, or CT scans of inadequate quality.
- Bronchiectasis.
- Lung cancer.
- Collagen vascular disorders.
- Interstitial lung diseases.
- Severe congestive heart failure.
- Severe renal failure requiring dialysis.
- Hospital-acquired pneumonia.
- Pulmonary thromboembolism (Fig. 2).

### Statistical analysis

All statistical analyses were performed using SPSS software (version 27.0; IBM Corp., Chicago, IL, USA). The distribution of continuous variables was assessed using the Kolmogorov–Smirnov test, along with visual methods including histograms, Q–Q plots, and boxplots, and by evaluating skewness and kurtosis values. Continuous variables with a normal distribution were expressed as mean ± standard deviation (SD), whereas non-normally distributed variables were presented as median with interquartile range (IQR). Categorical variables were summarized as frequencies and percentages. Comparisons between categorical variables were performed using Pearson’s chi-square test or Fisher’s exact test, as appropriate. For continuous variables, Student’s t-test was used for normally distributed data, while the Mann–Whitney U test was applied for non-normally distributed data. A two-sided p value < 0.05 was considered statistically significant. To evaluate the effect of emphysema on both dependent variables, univariable and subsequently multivariable logistic regression analyses were performed. Variables with a p value < 0.20 in the univariable analysis were included in the multivariable logistic regression model. The Hosmer-Lemeshow goodness-of-fit test evaluated the model fit, with *p* > 0.05 indicating a good fit.

## Results

### Study population

A total of 450 patients with COPD admitted to the chest diseases clinic with a diagnosis of pneumonia were screened. Of these, 135 patients met the study inclusion criteria and were divided into two groups: those with emphysema (*n* = 71, 53%) and those without emphysema (*n* = 64, 47%). The study flowchart is presented in Fig. 2.

Across all patients, the median interval between CT imaging and hospitalization was 33 days (IQR − 75 to 172.5), with a range from − 420 to 378 days.

### Demographic and clinical characteristics

The mean age was similar between the two groups. The proportion of male patients was significantly higher in the emphysema group than in the non-emphysema group (95.8% vs. 81.3%, *p* = 0.007). The proportion of current or former smokers was also significantly higher in the emphysema group (98.6% vs. 84.4%, *p* = 0.003) (Table 1). Sputum production was more frequent in the non-emphysema group compared with the emphysema group (73.4% vs. 56.3%, *p* = 0.038) (Table 1).

**Table 1:** Demographic and clinical characteristics of COPD patients with pneumonia according to emphysema status

	Total (*n* = 135)	Emphysema (*n* = 71)	No Emphysema (*n* = 64)	*p*
Age (years), mean (± SD)	72.79 (9.43)	73.23 (8.51)	72.31 (10.39)	0.58
*Gender*,* n (%)*				***0.007***
Male	120 (88.9)	68 (95.8)	52 (81.3)	
*Smoking history (n*,* %)*				***0.003***
Current/Former	124 (91.9)	70 (98.6)	54 (84.4)	
Never	11 (8.1)	1 (1.4)	10 (15.6)	
*Pack-years of cigarette smoking*				
* Median (IQR)*	50 (40–60)	50 (40–60)	50 (38–60)	0.83
*Comorbidities (n*,* %)*				
Diabetes	28 (20.7)	12 (16.9)	16 (25)	0.24
Hypertension	53 (39.3)	27 (38)	26 (40.6)	0.75
Cardiovascular diseases	42(31.1)	23 (32.4)	19 (29.7)	0.73
Peripheral artery disease	4 (3)	1 (1.4)	3 (4.7)	0.34
*Baseline Symptoms (n*,* %)*				
Fever	8 (5.9)	5 (7)	3 (4.7)	0.72
Dyspnea	132 (97.8)	69 (97.2)	63 (98.4)	1.00
Sputum	87 (64.4)	40 (56.3)	47 (73.4)	***0.038***
Pleuritic chest pain	33 (24.4)	10 (14.1)	23 (35.9)	***0.003***
Cough	65 (48.1)	39 (54.9)	26 (40.6)	0.09
*Pneumonia location (n*,* %)*				
Left lung	42 (31.1)	18 (25.4)	24 (37.5)	0.13
Right lung	52 (38.5)	27 (38)	25 (39.1)	0.90
Bilateral	41 (30.4)	26 (36.6)	15 (23.4)	0.09
*Parapneumonic effusion (n*,* %)*				0.11
No	99 (73.3)	48 (67.6)	51 (79.7)	
Yes	36 (26.7)	23 (32.4)	13 (20.3)	
*Pneumonia scoring systems*				
PSI, mean (± SD)	96.49 (25.88)	96.19 (23.46)	96.81 (28.48)	0.88
CURB-65, median (IQR)	1.0 (1.0–2.0)	1.00 (1.0–2.0)	2.00 (1.0–2.0)	0.15
*Regular medication (n*,* %)*				
* LABA/ICS*	55 (79.7)	32(84.2)	23(74.2)	0.30
* SABA/SAMA*	20(19.8)	14 (29.8)	6 (11.1)	0.019
* LAMA*	31(30.7)	20 (42.6)	11(20.4)	**0.016**
* LAMA+LABA + ICS*	4 (5.9)	1(2.6)	3(10)	0.31
*Hospital use of nebulized CS and/or systemic CS (n*,* %)*				0.98
Yes	99 (73.3)	52(73.2)	47 (73.4)	
No	36 (26.7)	19 (26.8)	17 (26.6)	
Antibiotics prior to admission, (n %)	43 (31.9)	25 (35.2)	18(28.1)	0.38
*Oxygen alone/Ventilation support (n*,* %)*				
Room air	23 (17)	10 (14.1)	13 (20.3)	0.33
Nasal oxygen	84 (62.2)	46 (64.8)	38 (59.4)	0.51
NIMV	28 (20.7)	15 (21.1)	13 (20.3)	0.90
*Length of Hospital Stay (days)*				
*Median (IQR)*	8 (7–11)	9 (7–14)	7.5 (6–10)	***0.010***
*Clinical outcomes (n*,* %)*				
Discharge	118 (87.4)	60 (84.5)	58 (90.6)	0.28
Intensive Care Unit admission	15 (11.1)	10 (14.1)	5 (7.8)	0.24
In-hospital mortality	3 (2.2)	2 (2.8)	1 (1.6)	1.0

*Abbreviations: CS* corticosteroid, *CURB-65* confusion, urea, respiratory rate, blood pressure, age ≥ 65 (Each criterion was scored with 1 point), *IQR* interquartile range, *LAMA* long-acting muscarinic antagonist, *LABA/ICS* long-acting beta2-agonist/inhaled corticosteroid, *NIMV* non-invasive mechanical ventilation, *PSI* pneumonia severity index, *SD* standard deviation, *SABA/SAMA* short- acting beta2-agonist/short- acting muscarinic antagonist

Regarding the anatomical localization of pneumonia among all patients, the right lung was the most commonly involved site (52 patients, 38.5%), followed by the left lung (42 patients, 31.1%) and bilateral involvement (41 patients, 30.4%). Parapneumonic effusion was present in 36 patients (26.7%). There were no significant differences between the groups in terms of pneumonia localization or the presence of pleural effusion (*p* > 0.05 for all comparisons) (Table 1).

Various scores were used to assess pneumonia severity and were compared between groups. As shown in Table 1, the CURB-65 and PSI scores at hospital admission were comparable between the emphysema and non-emphysema groups (PSI: *p* = 0.88; CURB-65: *p* = 0.15), indicating that pneumonia severity was balanced across groups.

Comparison of chronic and in-hospital bronchodilator treatments between the two groups showed that LAMA use was more frequent in the emphysema group. Although the use of nasal oxygen and NIMV during hospitalization was more frequent in patients with emphysema than in those without emphysema, these differences did not reach statistical significance. Discharge rates, need for ICU admission, and in-hospital mortality were similar between the two groups. The median length of hospital stay was significantly longer in the emphysema group than in the non-emphysema group (9 days [IQR 7–14] vs. 7 days [IQR 6–10], *p* = 0.010) (Table 1).

The demographic and clinical characteristics of COPD patients with pneumonia according to emphysema status are summarized in Table 1, which is presented at the end of the manuscript due to its length.

### Emphysema subtypes and severity

**Fig. 3:**
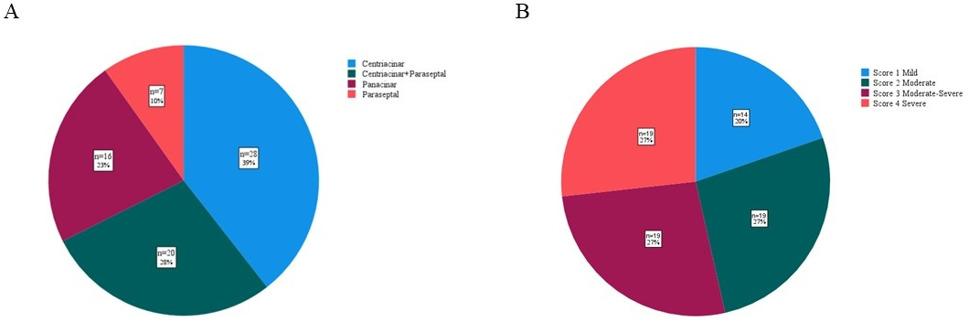
Emphysema Types and Scoring. **A** Emphysema Types. **B** Emphysema Scoring

Among the 71 patients with emphysema, the most common subtype was centriacinar emphysema (*n* = 28, 39%), followed by mixed centriacinar plus paraseptal emphysema (*n* = 20, 28%), panacinar emphysema (*n* = 16, 23%), and paraseptal emphysema (*n* = 7, 10%) (Fig. 3a).

**Fig. 4:**
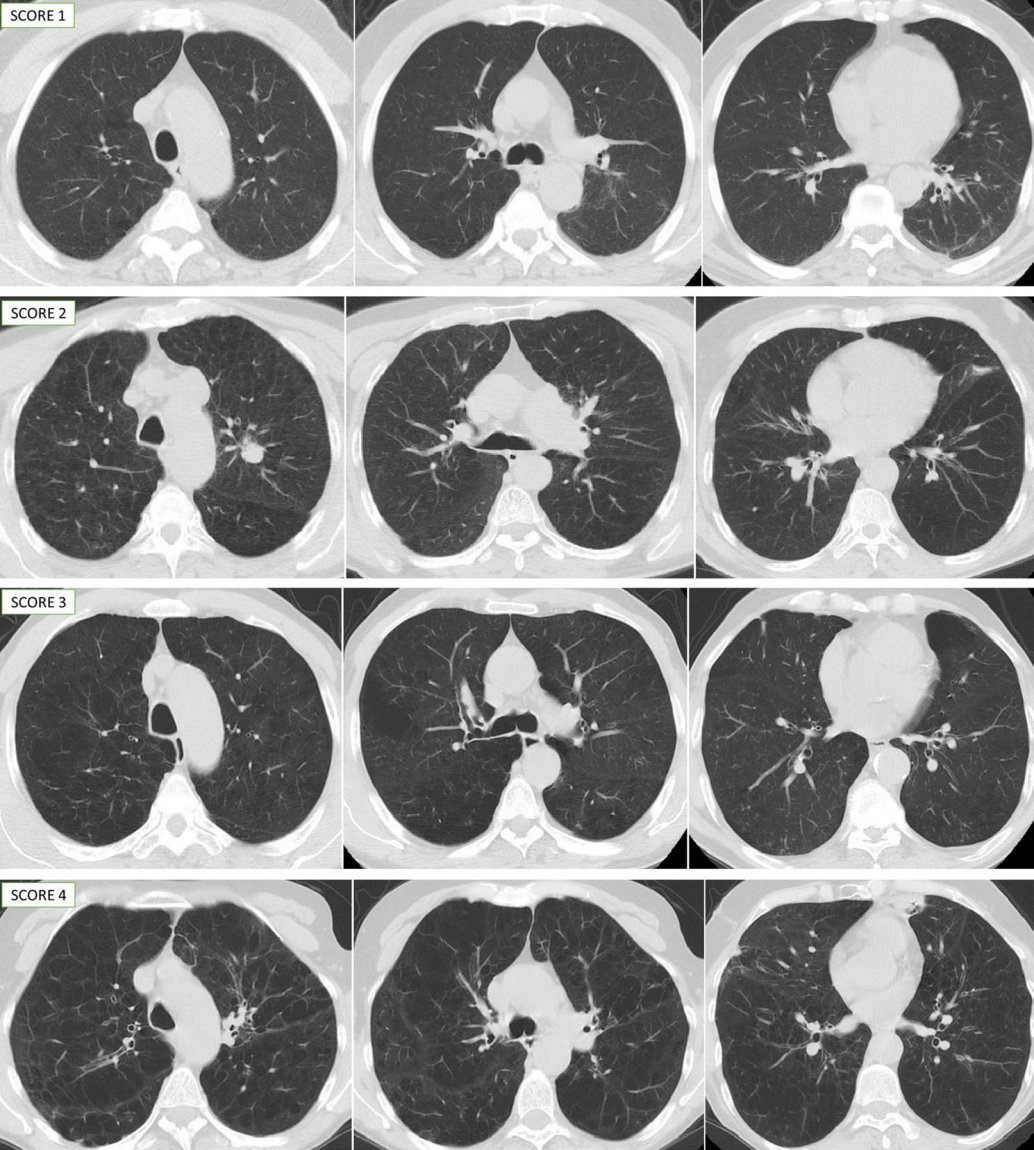
Example thoracic CT images of emphysema scoring

According to the emphysema scoring system, 14 patients had mild emphysema (score 1), 19 had moderate emphysema (score 2), 19 had moderate-to-severe emphysema (score 3), and 19 had severe emphysema (score 4). The distribution of emphysema severity scores is shown in Fig. 3b, and representative thoracic CT images corresponding to each emphysema grade are presented in Fig. 4.

### Microbiological analysis

Sputum cultures from 41 patients showed normal respiratory flora, whereas a pathogenic microorganism was isolated in 6 patients. The identified pathogens included Klebsiella pneumoniae (*n* = 1), Acinetobacter baumannii (*n* = 2), Pseudomonas aeruginosa (*n* = 1), Stenotrophomonas maltophilia (*n* = 1), and Gram-positive cocci (*n* = 1). Blood cultures were performed in 23 patients, and no growth was observed in any of them. In the two patients with sputum cultures positive for Stenotrophomonas maltophilia and Acinetobacter baumannii, corresponding blood cultures were also negative.

### Biomarkers and laboratory findings

**Fig. 5:**
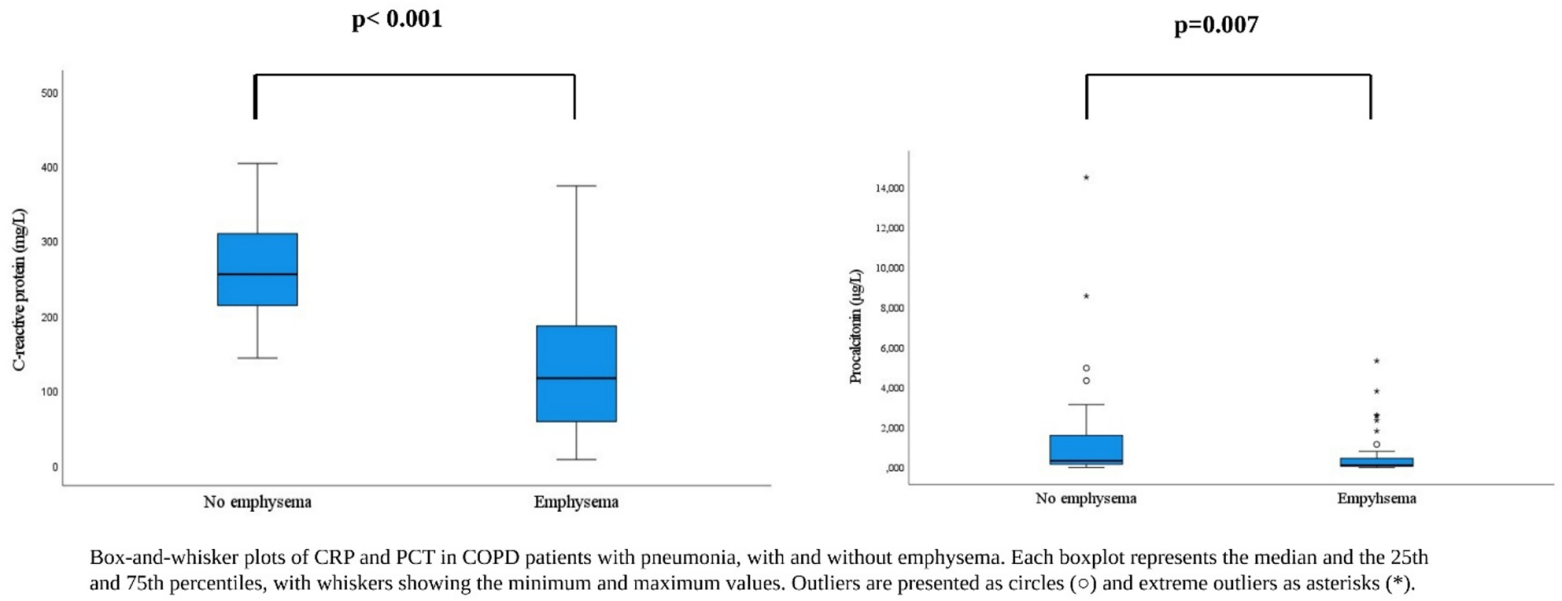
Box-and-whisker plots of CRP and PCT in COPD patients with pneumonia, with and without emphysema

Comparison of inflammatory biomarkers revealed that median CRP levels were significantly lower in the emphysema group than in the non-emphysema group (116 mg/L IQR [56–187] vs. 254 mg/L IQR [213–310], *p* < 0.001 respectively). Similarly, median PCT levels were significantly lower in the emphysema group compared with the non-emphysema group (0.132 µg/L IQR [0.079–0.536] vs. 0.356 µg/L IQR [0.171–1.660], *p* = 0.007 respectively) (Table 2; Fig. 5).

**Table 2:** Initial laboratory parameters COPD patients with pneumonia according to emphysema status

	Total (*n* = 135)	Emphysema (*n* = 71)	No Emphysema (*n* = 64)	*p*
Hematological values				
WBC (x10³/µL), median (IQR)	12.62 (9.60–17.52)	12.02 (8.51–16.93)	14.27 (10.99–17.73)	***0.036***
Neutrophils (x10³/µL), median (IQR)	10.08 (7.48–14.20)	9.26 (6.70–13.90)	11.52 (8.54–14.27)	***0.035***
Lymphocyte (x10³/µL), median (IQR)	1.39 (0.76–1.70)	1.35 (0.70–1.69)	1.43 (0.80–1.77)	0.388
Platelet (x10³/µL), median (IQR)	270 (204–353)	278 (221–381)	250 (198–303)	**0.042**
Eosinophil (x10³/µL), median (IQR)	0.07(0.0–0.20)	0.09 (0.01–0.20)	0.03 (0.0–0.13)	0.063
*Biochemistry values*				
Urea (mg/dL), median (IQR)	41 (30–52)	41 (29–51)	42 (30–53.75)	0.785
Creatinine (mg/dL), median (IQR)	0.90 (0.75–1.09)	0.83 (0.75–0.97)	1.01(0.76–1.24)	***0.005***
Sodium (mmol/L), median (IQR)	137 (134–140)	137 (135–140)	136 (133.25–140)	0.434
GFR (ml/min/1.73 m²), median (IQR)	87.20 (23–270)	91.90 (80.90–106.80)	74.75 (56.67–103.85)	0.001
Potassium (mmol/L), median (IQR)	4.57 (4.20–4.98)	4.55 (4.20–4.99)	4.58(4.19–4.96)	0.785
*Inflammatory markers*				
CRP (mg/L), median (IQR)	210 (107–265)	116 (56–187)	254 (213–310)	***< 0.001***
PCT (µg/L), median (IQR)	0.235 (0.092–0.786)	0.132 (0.079–0.536)	0.356 (0.171–1.660)	***0.007***
NLR, median (IQR)	8.29 (4.97–14.09)	8.37 (3.73–13.51)	8.20 (5.76–14.69)	0.370

*Abbreviations: CRP* C-reactive protein, *GFR* glomerular filtration rate, *NLR* neutrophil – lymphocyte ratio, *PCT* procalcitonin, *WBC* white blood cell

Analysis of complete blood count parameters demonstrated significantly lower median WBC and neutrophil counts in the emphysema group compared with the non-emphysema group (WBC: 12.02 × 10³/µL IQR [8.51–16.93] vs. 14.27 × 10³/µL IQR [10.99–17.73], *p* = 0.036; neutrophils: 9.26 × 10³/µL IQR [6.7–13.9] vs. 11.52 × 10³/µL IQR [8.54–14.27], *p* = 0.035) (Table 2).

No significant differences were observed between the two groups in biochemical parameters, except for serum creatinine levels. Median creatinine was significantly lower in the emphysema group than in the non-emphysema group (0.83 mg/dL IQR [0.75–0.97] vs. 1.01mg/dL IQR [0.76–1.24], *p* = 0.005) (Table 2).

When all patients were stratified according to their GFR values, 97.8% were classified as Stage 1–3. Only three patients were in Stage 4, and no patients were in Stage 5. Patients in Stage 4 were excluded from the procalcitonin (PCT) analysis, as PCT levels may be affected by impaired renal function.

### Variables associated with low CRP (≤ 200 mg/L) levels: binary logistic regression analysis

In the binary logistic regression analysis conducted to evaluate factors associated with low CRP levels (≤ 200 mg/L), the presence of emphysema was found to be strongly associated in both univariable and multivariable logistic regression analyses. In the univariable analysis, the presence of emphysema significantly increased the likelihood of low CRP (OR 44.053; 95% CI 15.018–129.224; *p* < 0.001). This association remained significant in the multivariable model after adjusting for potential confounding variables (OR 54.274; 95% CI 14.545–202.526; *p* < 0.001), indicating that emphysema is an independent predictor.

When other variables were examined, each one-unit increase in GFR was independently associated with a 2% higher likelihood of low CRP (OR 1.021; 95% CI 1.002–1.040; *p* = 0.026). In addition, antibiotic use within the last month prior to hospital admission was found to be significantly associated with low CRP levels both in the univariable analysis (OR 2.491; 95% CI 1.186–5.232; *p* = 0.016) and in the multivariable analysis (OR 4.278; 95% CI 1.199–15.264; *p* = 0.025) (Table 3).

**Table 3:** Variables associated with low CRP (≤ 200 mg/L) levels: binary logistic regression analysis

Characteristic								
	Univariable logistic regression				Multivariable logistic regression			
	OR	95%CI		p	OR	95%CI		p
Emphysema (yes vs. no)	44.053	15.018	129.224	***< 0.001***	54.274	14.545	202.526	***< 0.001***
Age, year	1.024	0.987	1.062	0. 209				
Gender (men vs. female)	2.488	0.750	8.254	0.136	0.377	0.024	5.899	0.487
Smoking status (ever vs. never)	4.015	0.833	19.350	0.083	3.551	0.101	124.295	0.485
Pack-years of cigarette smoking	1.00	0.98	1.02	0.66				
Diabetes	0.888	0.383	2.055	0.781				
Cardiovascular diseases	1.003	0.482	2.086	0.993				
Systemic corticosteroid use	1.054	0.534	2.083	0.879				
GFR	1.023	1.010	1.035	***< 0.001***	1.021	1.002	1.040	***0.026***
CURB-65	1.375	0.854	2.216	0.190	1.221	0.560	2.665	0.615
Antibiotics prior to admission (yes vs. no)	2.491	1.186	5.232	0.***016***	4.278	1.199	15.264	***0.025***
Model summary								
* R²(Nagelkerke)*						0.657		
* R² (Cox and Snell)*						0.491		
*Hosmer-Lemeshow significance*						0.283		

Variables with *p* < 0.20 in univariable analysis were entered into the multivariable regression model

*Abbreviations: CI* confidence interval, *CURB-65* confusion, urea, respiratory rate, blood pressure, age ≥ 65 (Each criterion was scored with 1 point), *GFR* glomerular filtration rate, *OR* odds ratio

Among the other variables, smoking status was associated with low CRP in the univariable analysis (OR 4.015; *p* = 0.083) but was no longer significant in the multivariable model (*p* = 0.485**).** Age, sex, pack-years of cigarette smoking, diabetes, cardiovascular diseases, systemic corticosteroid use, and CURB-65 score did not show a significant association with low CRP in either analysis. Model fit was assessed using the Hosmer–Lemeshow test and found to be good (*p* = 0.283). The model’s explanatory power was Nagelkerke R² = 0.657 and Cox & Snell R² = 0.491 (Table 3).

### Variables associated with low PCT (< 0.1 µg/L) levels: binary logistic regression analysis

In the binary logistic regression analysis conducted to evaluate factors associated with low PCT levels (< 0.1 µg/L), only the presence of emphysema was found to be associated with low PCT in both the univariable (OR 4.371; 95% CI 1.441–13.263; *p* = 0.009) and multivariable logistic regression analyses (OR 3.694; 95% CI 1.145–11.921; *p* = 0.029). Model fit was assessed using the Hosmer–Lemeshow test and was found to be good (*p* = 0.592). The explanatory power of the model was indicated by Nagelkerke R² = 0.221 and Cox & Snell R² = 0.151 (Table 4).

**Table 4:** Variables associated with low PCT (< 0.1 µg/L) levels: binary logistic regression analysis

Characteristic								
	Univariable logistic regression				Multivariable logistic regression			
	OR	95%CI		p	OR	95%CI		p
Emphysema (yes vs. no)	4.371	1.441	13.263	***0.009***	3.694	1.145	11.921	***0.029***
Age, year	0.986	0.935	1.041	0.616				
Gender (men vs. female)	1.556	0.305	7.935	0.595				
Smoking status (ever vs. never)	1.517	0.161	14.332	0.716				
Pack-years of cigarette smoking	1.00	0.972	1.029	0.981				
Diabetes	0.660	0.194	2.245	0.506				
Cardiovascular diseases	0.741	0.254	2.166	0.584				
Systemic corticosteroid use	1.164	0.447	3.032	0.756				
GFR	1.012	0.999	1.026	0.075	1.007	0.993	1.022	0.315
CURB-65	0.491	0.250	0.966	***0.039***	0.556	0.263	1.176	0.125
Antibiotics prior to admission (yes vs. no)	2.423	0.859	6.834	0.094	2.213	0.727	6.744	0.162
Model summary								
*R²(Nagelkerke)*						0.221		
*R² (Cox and Snell)*						0.151		
*Hosmer-Lemeshow significance*						0.592		

Variables with *p* < 0.20 in univariable analysis were entered into the multivariable regression model

*Abbreviations: CI* confidence interval, *CURB-65* confusion, urea, respiratory rate, blood pressure, age ≥ 65 (Each criterion was scored with 1 point), *GFR* glomerular filtration rate, *OR* odds ratio

## Discussion

In this study, we demonstrated that two of the most commonly used inflammatory biomarkers in the diagnosis of pneumonia, CRP and PCT, were significantly lower in patients with emphysema compared with those without emphysema. This difference remained consistent in both univariable and multivariable logistic regression analyses, supporting the independent association between emphysema and lower biomarker levels. Furthermore, this association appeared to be more pronounced for CRP than for PCT, suggesting that CRP may be more strongly influenced by the presence of emphysema in this clinical context.

Consistent with previous studies, the emphysema with pneumonia in our cohort was predominantly male, and tobacco exposure was significantly more common than in the non-emphysema group [23]. The most plausible explanation for this finding is the higher prevalence of smoking among men in this population, as smoking leads to progressive structural lung damage and accelerated alveolar aging, which are central to emphysema pathogenesis [24].

COPD has been reported as an independent risk factor for pneumonia, and the coexistence of these two conditions has been shown to adversely affect prognosis [25, 26]. In a study of 1,032 patients hospitalized with CAP, COPD was associated with a 1.91-fold increased risk of severe pneumonia [25]. Similarly, a recent meta-analysis of thirteen studies demonstrated that when pneumonia coexists with COPD, both short- and long-term mortality increase and hospital length of stay is prolonged [27]. Three major mechanisms have been proposed to explain pneumonia development in COPD: alterations in the lung microbiome, impaired pulmonary immune responses, and pathogen virulence [7]. The mucosal surfaces of the lungs in patients with COPD are continuously exposed to microbial pathogens, which may precipitate pneumonia in susceptible individuals [28]. Several studies indicate that the emphysema phenotype of COPD is associated with an increased risk of pneumonia and worse prognosis. Hong et al. evaluated multiple parameters for pneumonia risk and identified only two independent risk factors: the presence of emphysema and post-bronchodilator FEV₁%. Furthermore, when emphysema extent was classified by CT as mild, moderate, or severe, the incidence of subsequent pneumonia over time was highest in patients with severe emphysema [29]. Similarly, Eom et al. classified patients based on the presence or absence of emphysema and reported that emphysema increased the risk of developing severe pneumonia by approximately 3.5-fold [30]. The higher pneumonia risk observed in emphysema may be related to greater tobacco exposure in this population. Structural and functional lung damage caused by smoking accelerates emphysema development and increases susceptibility to infection, thereby influencing pneumonia risk. In a cellular-level study using electron microscopy, Vij et al. demonstrated that smoking inhibits autophagy, resulting in increased susceptibility to infections [24]. In addition, surfactant—which plays a critical role in pulmonary immunity—decreases with tobacco exposure, and surfactant deficiency contributes to emphysema formation, providing another potential explanation for the increased pneumonia risk associated with emphysema [31].

Pneumonia is characterized by infection and inflammation of the lung parenchyma, and the most commonly used diagnostic infection markers include CRP, PCT and WBC/neutrophil counts [4]. Although CRP has limited specificity, it is a highly sensitive marker of inflammation and tissue damage [13]. There are a limited number of studies in the literature demonstrating different inflammatory responses in the presence of emphysema [23]. The effect of emphysema on biomarkers in cases of coexisting emphysema and pneumonia has not been sufficiently investigated. In one of the few studies using quantitative CT analysis to assess emphysema extent, small-airway disease, and bronchial wall thickness, greater emphysema severity was associated with a lower systemic inflammatory response, reflected by reduced CRP (β = −0.34, *p* < 0.001) and fibrinogen levels (β = −0.28, *p* = 0.003) [32]. However, some studies have reported contrasting results, with higher biomarker levels observed in patients with emphysema. In one such study, emphysema scoring was performed using a method similar to that of the present study, and higher levels of the same biomarkers were reported in the emphysema group; however, that study included patients without COPD in the emphysema cohort [23]. In another study, oxidative stress markers and fibrinogen levels were higher in emphysema patients than in non-emphysema patients, but biomarkers were assessed only during the stable phase of COPD, and detailed emphysema scoring was not performed [33]. In our study, unlike other studies, infection biomarkers were investigated in the coexistence of emphysema and pneumonia. CRP levels were shown to be lower in COPD patients with emphysema. The presence of emphysema remained significant in multivariable analysis after adjustment for potential confounding variables such as smoking, diabetes, chronic heart disease, GFR, and prior antibiotic use, and was shown to be an independent predictor of lower CRP levels. However, the relatively large odds ratio observed for CRP should be interpreted with caution. While this finding reflects a strong association between emphysema status and CRP levels, the wide confidence interval indicates a degree of uncertainty in the estimate. This may be partly explained by the moderate sample size and the marked separation between groups observed in the dataset. Therefore, the magnitude of the effect should not be overinterpreted, and future studies using larger cohorts and alternative modeling strategies are warranted.

PCT is, after CRP, another biomarker most frequently used in the diagnosis of pneumonia [13]. However, many studies in the literature indicate that PCT is more useful in the management of antibiotic therapy particularly in decisions regarding initiation and discontinuation rather than for its diagnostic value [13, 34]. The fact that PCT is sensitive only to bacterial infections, that this sensitivity ranges widely from 38% to 91%, that it can be low in severe infections, and that it can be affected by impaired kidney function, limits its use for diagnosis [35]. Although there is no recommended safe cut-off value for pneumonia diagnosis, a value of < 0.1 ng/mL has been reported to exclude infection and indicate that antibiotic treatment is not required [21]. In our study, when the cut-off value for PCT was set at 0.1 ng/mL, the presence of emphysema alone was found to significantly affect low PCT levels, independent of antibiotic use and GFR. This effect was less pronounced compared to CRP. In our study, CRP and PCT were analyzed using clinically established cut-off values; however, we acknowledge that dichotomization of continuous variables may lead to loss of information and reduced statistical power.

The underlying mechanism responsible for lower infection biomarker levels in patients with emphysema remains unclear. Several potential explanations may be proposed. First, extensive destruction of the lung parenchyma in emphysema may impair the ability to mount a robust systemic inflammatory response. Second, differences in mucus production may contribute to this finding. Increased mucus production is a characteristic feature of chronic bronchitis rather than emphysema. Persistent mucus accumulation can enhance airway inflammation, promote infection, and worsen airflow obstruction, potentially leading to proliferation of pathogenic bacteria [28].

Although the differences did not reach statistical significance, patients with emphysema in our cohort tended to have more frequent bilateral pneumonia, a higher incidence of pleural effusion, greater use of nasal oxygen and NIMV, and more frequent transfer to the ICU compared with patients without emphysema. This trend, consistent with previous reports, suggests that pneumonia prognosis may be worse in patients with emphysema [23, 29, 30].

This study has several limitations. First, the lack of pulmonary function test data is a limitation of this study, as spirometric data were not available in the electronic database.

However, all diagnoses were made by specialist physicians at a tertiary referral chest diseases training and research hospital based on spirometric assessments, supporting diagnostic accuracy. Second, the retrospective and single-center design limits generalizability. Another limitation of our study is the lack of data on prior exacerbation history and vaccination status, which may have influenced inflammatory responses and clinical outcomes. Additionally, the absence of comprehensive microbiological data, including viral testing, is another limitation, as it restricts our ability to attribute differences in biomarkers to specific etiologies.

Nevertheless, key strengths of the study include the use of thoracic imaging in all patients, enabling accurate diagnosis of emphysema and pneumonia, and detailed classification and scoring of emphysema subtypes by experienced clinicians.

## Conclusion

In patients with an emphysema phenotype of COPD who develop community-acquired pneumonia, commonly used infection biomarkers such as CRP, PCT, WBC, and neutrophil counts may be lower compared to COPD patients without emphysema. CT-defined emphysema was associated with reduced biomarker levels at presentation and longer hospital stay, suggesting a potential association between emphysema status and the systemic inflammatory response in COPD.

These findings indicate that the inflammatory response to pneumonia may differ according to emphysema phenotype. However, causality cannot be inferred from this observational study, and the results should be interpreted cautiously. Taken together, these findings are hypothesis-generating and primarily serve to inform future research directions. Future prospective, multicenter studies are needed to confirm these observations and further explore their biological and clinical relevance.

## Data Availability

The datasets generated and/or analyzed during the current study are available from the corresponding author on reasonable request.

## Abbreviations

COPD: Chronic Obstructive Pulmonary Disease
CRP: C-Reactive Protein
GFR: Glomerular Filtration Rate
PCT: Procalcitonin
WBC: White Blood Cell
CAP: Community-Acquired Pneumonia
ICU: Intensive Care Unit
CT: Computed Tomography
HRCT: High-Resolution Computed Tomography
NIMV: Non-Invasive Mechanical Ventilation
ICD: International Classification of Diseases
PACS: Picture Archiving and Communication System
PSI: Pneumonia Severity Index
CURB-65: Confusion, Urea, Respiratory Rate, Blood Pressure, Age ≥ 65
IQR: Interquartile Range

## References

[1] World Health Organization. Chronic obstructive pulmonary disease (COPD). Eastern Mediterranean Regional Office, WHO. Available from:https://www.emro.who.int/health-topics/chronic-obstructive-pulmonary-disease-copd/. Accessed 24 Dec 2025.

[2] Global Initiative for Chronic Obstructive Lung Disease (GOLD). Global strategy for the diagnosis, management, and prevention of chronic obstructive pulmonary disease: 2024 report. 2024. Available from: https://goldcopd.org/2024-gold-report/. Accessed 22 May 2026.

[3] Snider GL, Kleinerman J, Thurlbeck WM, Bengali ZH. The definition of emphysema: report of a National Heart, Lung and Blood Institute, Division of Lung Diseases Workshop. Am Rev Respir Dis. 1985;132(1):182–5. 10.1164/arrd.1985.132.1.182. 4014865 10.1164/arrd.1985.132.1.182

[4] Musher DM, Thorner AR. Community-acquired pneumonia. N Engl J Med. 2014;371(17):1619–28. 10.1056/NEJMra1312885. 25337751 10.1056/NEJMra1312885

[5] World Health Organization. WHO reveals leading causes of death and disability worldwide: 2000–2019. December 2020. Available from:https://www.who.int/news/item/09-12-2020-who-reveals-leading-causes-of-death-and-disability-worldwide-2000-2019. Accessed 24 Dec 2025.

[6] Ramirez JA, Wiemken TL, Peyrani P, Arnold FW, Kelley R, Mattingly WA, et al. Adults hospitalized with pneumonia in the United States: incidence, epidemiology, and mortality. Clin Infect Dis. 2017;65(11):1806–12. 10.1093/cid/cix647. 29020164 10.1093/cid/cix647

[7] Cavallazzi R, Ramirez J. Community-acquired pneumonia in chronic obstructive pulmonary disease. Curr Opin Infect Dis. 2020;33(2):173–81. 10.1097/QCO.0000000000000639. 32022741 10.1097/QCO.0000000000000639

[8] Jen R, Rennard SI, Sin DD. Effects of inhaled corticosteroids on airway inflammation in chronic obstructive pulmonary disease: a systematic review and meta-analysis. Int J Chron Obstruct Pulmon Dis. 2012;7:587–95. 10.2147/COPD.S32765. 23055709 10.2147/COPD.S32765 PMC3459653

[9] Brusselle GG, Demoor T, Bracke KR, Brandsma CA, Timens W. Lymphoid follicles in (very) severe COPD: beneficial or harmful? Eur Respir J. 2009;34:219–30. 10.1183/09031936.00150208. 19567605 10.1183/09031936.00150208

[10] Crisafulli E, Menendez R, Huerta A, et al. Systemic inflammatory pattern of patients with community-acquired pneumonia with and without COPD. Chest. 2013;143(4):1009–17. 10.1378/chest.12-1684. 23187314 10.1378/chest.12-1684

[11] Gutierrez P, Closa D, Piner R, Bulbena O, Menendez R, Torres A. Macrophage activation in exacerbated COPD with and without community-acquired pneumonia. Eur Respir J. 2010;36(2):285–91. 10.1183/09031936.00118909. 20032016 10.1183/09031936.00118909

[12] Jiang HL, Chen HX, Liu W, Fan T, Liu GJ, Mao B, et al. Is COPD associated with increased mortality and morbidity in hospitalized pneumonia? A systematic review and meta-analysis. Respirology. 2015;20:1046–54. 10.1111/resp.12597. 26177049 10.1111/resp.12597

[13] Krüger S, Welte T. Biomarkers in community-acquired pneumonia. Expert Rev Respir Med. 2012;6(2):203–14. 10.1586/ers.12.6. 22455492 10.1586/ers.12.6

[14] Bafadhel M, Clark TW, Reid C, et al. Procalcitonin and C-reactive protein in hospitalized adult patients with community-acquired pneumonia or exacerbation of asthma or COPD. Chest. 2011;139(6):1410–8. 10.1378/chest.10-1747. 21030489 10.1378/chest.10-1747 PMC3109646

[15] Titova E, Christensen A, Henriksen AH, et al. Comparison of procalcitonin, C-reactive protein, white blood cell count and clinical status in diagnosing pneumonia in patients hospitalized with acute exacerbations of COPD: a prospective observational study. Chron Respir Dis. 2019;16:1–9. 10.1177/1479972318769762. 10.1177/1479972318769762 PMC6302976 29848051

[16] Cottin V, Nunes H, Brillet PY, et al. Combined pulmonary fibrosis and emphysema: a distinct under recognised entity. Eur Respir J. 2005;26(4):586–93. 10.1183/09031936.05.00021005. 16204587 10.1183/09031936.05.00021005

[17] Goddard PR, Nicholson EM, Laszlo G, Watt I. Computed tomography in pulmonary emphysema. Clin Radiol. 1982;33(4):379–87. 10.1016/s0009-9260(82)80301-2. 7083738 10.1016/s0009-9260(82)80301-2

[18] Fine MJ, Auble TE, Yealy DM, et al. A prediction rule to identify low-risk patients with community-acquired pneumonia. N Engl J Med. 1997;336(4):243–50. 10.1056/NEJM199701233360402. 8995086 10.1056/NEJM199701233360402

[19] Lim WS, van der Eerden MM, Laing R. Defining community acquired pneumonia severity on presentation to hospital: an international derivation and validation study. Thorax. 2003;58(5):377–82. 10.1136/thorax.58.5.377. 12728155 10.1136/thorax.58.5.377 PMC1746657

[20] Ruiz-González A, Utrillo L, Bielsa S, Falguera M, Porcel JM. The diagnostic value of serum C-Reactive Protein for identifying pneumonia in hospitalized patients with acute respiratory symptoms. J Biomarkers. 2016;2016:2198745. 10.1155/2016/2198745. 10.1155/2016/2198745 PMC5004021 27610265

[21] Huang DT, Angus DC, Chang CCH, Doi Y, Fine MJ, Kellum JA, et al. Design and rationale of the Procalcitonin Antibiotic Consensus Trial (ProACT), a multicenter randomized trial of procalcitonin antibiotic guidance in lower respiratory tract infection. BMC Emerg Med. 2017;17(1):25. 10.1186/s12873-017-0138-1. 28851296 10.1186/s12873-017-0138-1 PMC5576372

[22] National Kidney Foundation. K/DOQI clinical practice guidelines for chronic kidney disease: evaluation, classification, and stratification. Am J Kidney Dis. 2002;39(2 Suppl 1):S1-266. 11904577

[23] Seo H, Cha SI, Shin KM, Lim JK, Lee WK, Park JE, et al. Clinical relevance of emphysema in patients hospitalized with community-acquired pneumonia: clinical features and prognosis. Clin Respir J. 2021;15(7):826–34. 10.1111/crj.13370. 33826807 10.1111/crj.13370

[24] Vij N, Chandramani-Shivalingappa P, Van Westphal C, Hole R, Bodas M. Cigarette smoke-induced autophagy impairment accelerates lung aging, COPD-emphysema exacerbations and pathogenesis. Am J Physiol Cell Physiol. 2018;314(1):C73-87. 10.1152/ajpcell.00110.2016. 27413169 10.1152/ajpcell.00110.2016 PMC5866380

[25] Ishiguro T, Takayanagi N, Yamaguchi S, Yamakawa H, Nakamoto K, Takaku Y, et al. Etiology and factors contributing to the severity and mortality of community-acquired pneumonia. InternMed. 2013;52(3):317–24. 10.2169/internalmedicine.52.8830. 10.2169/internalmedicine.52.8830 23370738

[26] De Miguel-Díez J, López-de-Andrés A, Hernández-Barrera V, et al. Impact of COPD on outcomes in hospitalized patients with community-acquired pneumonia: analysis of the Spanish national hospital discharge database (2004–2013). Eur J InternMed. 2017;43:69–76. 10.1016/j.ejim.2017.06.008. 10.1016/j.ejim.2017.06.008 28615117

[27] Zheng F, WangX. Effect of pneumonia on the outcomes of acute exacerbation of chronic obstructive pulmonary disease: a systematic review and meta-analysis. BMC PulmonaryMedicine. 2024;24(1):496. 10.1186/s12890-024-03305-1. 10.1186/s12890-024-03305-1 PMC11462751 39385140

[28] Restrepo MI, Sibila O, Anzueto A. Pneumonia in patients with chronic obstructive pulmonary disease. Tuberc Respir Dis. 2018;81:187–97. 10.4046/trd.2018.0030. 10.4046/trd.2018.0030 PMC6030662 29962118

[29] Hong Y, Lee JS, Yoo KH, Lee JH, Kim WJ, Lim SY, et al. Implications of emphysema and lung function for the development of pneumonia in patients with chronic obstructive pulmonary disease. TubercRespirDis (Seoul). 2016;79(2):91–7. 10.4046/trd.2016.79.2.91. 10.4046/trd.2016.79.2.91 PMC4823189 27066086

[30] Eom JS, Song WJ, Yoo H, Jeong BH, Lee HY, Koh WJ, et al. Chronic obstructive pulmonary disease severity is associated with severe pneumonia. Ann Thorac Med. 2015;10(2):105–11. 10.4103/1817-1737.151441. 25829961 10.4103/1817-1737.151441 PMC4375738

[31] Betsuyaku T, Kuroki Y, Nagai K, Nasuhara Y, Nishimura M. Effects of ageing and smoking on SP-A and SP-D levels in bronchoalveolar lavage fluid. Eur Respir J. 2004;24(6):964–70. 10.1183/09031936.04.00064004. 15572540 10.1183/09031936.04.00064004

[32] Ostridge K, Williams NP, Kim V, Harden S, Bourne S, Clarke SC, et al. Relationship of CT-quantified emphysema, small airways disease and bronchial wall dimensions with physiological, inflammatory and infective measures in COPD. Respir Res. 2018;19(1):31. 10.1186/s12931-018-0734-y. 29458372 10.1186/s12931-018-0734-y PMC5819274

[33] Papaioannou AI, Mazioti A, Kiropoulos T, Tsilioni I, Koutsokera A, Tanou K, et al. Systemic and airway inflammation and the presence of emphysema in patients with COPD. Respir Med. 2010;104(2):275–82. 10.1016/j.rmed.2009.09.016. 19854037 10.1016/j.rmed.2009.09.016

[34] Simon L, Gauvin F, Amre DK, Saint-Louis P, Lacroix J. Serum procalcitonin and C-reactive protein levels as markers of bacterial infection: a systematic review and meta-analysis. Clin Infect Dis. 2004;39(2):206–17. 10.1086/421997. 15307030 10.1086/421997

[35] Metlay JP, Waterer GW, Long AC, Anzueto A, Brozek J, Crothers K, et al. Diagnosis and treatment of adults with community-acquired pneumonia. An official clinical practice guideline of the American Thoracic Society and Infectious Diseases Society of America. Am J Respir Crit Care Med. 2019;200(7):e45-67. 10.1164/rccm.201908-1581ST. 31573350 10.1164/rccm.201908-1581ST PMC6812437

